# Supplementary material for: IFNλ is a potent anti‐influenza therapeutic without the inflammatory side effects of IFNα treatment
Source: EMBO Mol Med. 2016 Aug 12;8(9):1099–112. doi: 10.15252/emmm.201606413 (PMC5009813; doi:10.15252/emmm.201606413)
Supplement: Supplementary file 1 — Expanded View Figures PDF [file EMMM-8-1099-s001.pdf]

Expanded View Figures

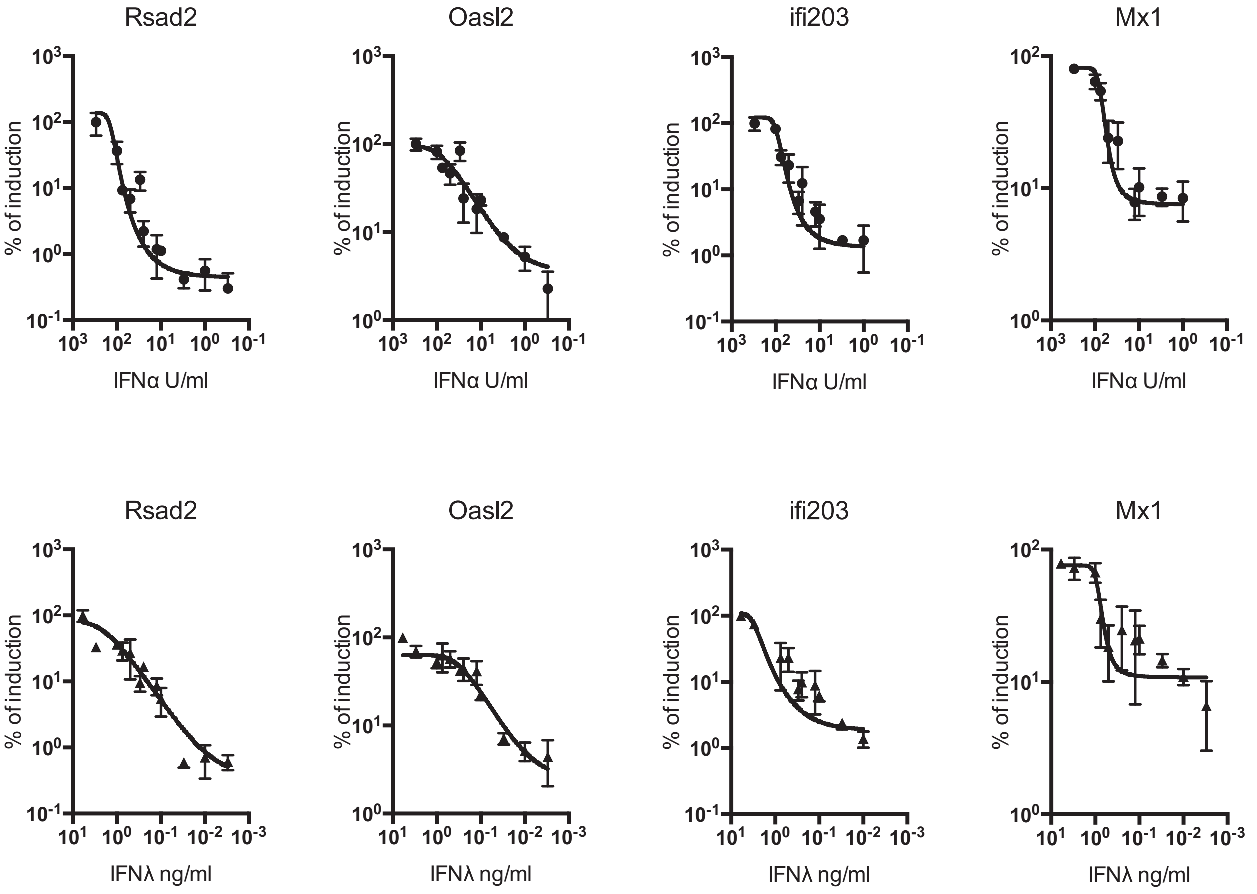

$$\frac{\text{EC50 IFN}\alpha\ \text{ISG induction}}{\text{EC50 IFN}\lambda\ \text{ISG induction}} = \text{Conversion Ratio}$$

Figure EV1. Comparison of epithelial responsiveness to IFNα4 and IFNλ2.
AEC cultures were stimulated for 4 h with IFNα4 (circles) or IFNλ2 (triangles), and induction of indicated ISGs was assessed by qPCR. Prism 6 software was used to perform a curve fit (Sigmoidal, 4PL) and generate a dose–response curve and a half-maximal effective concentration (EC_{50}) for each gene assessed for each treatment. A conversion ratio was then generated using the indicated formula for each ISG and a final conversion ratio was taken from the average of all ISGs assessed. Values represent means ± SEM. Data are pooled from two independent experiments where $n = 3–7$ per experiment.

© 2016 The Authors

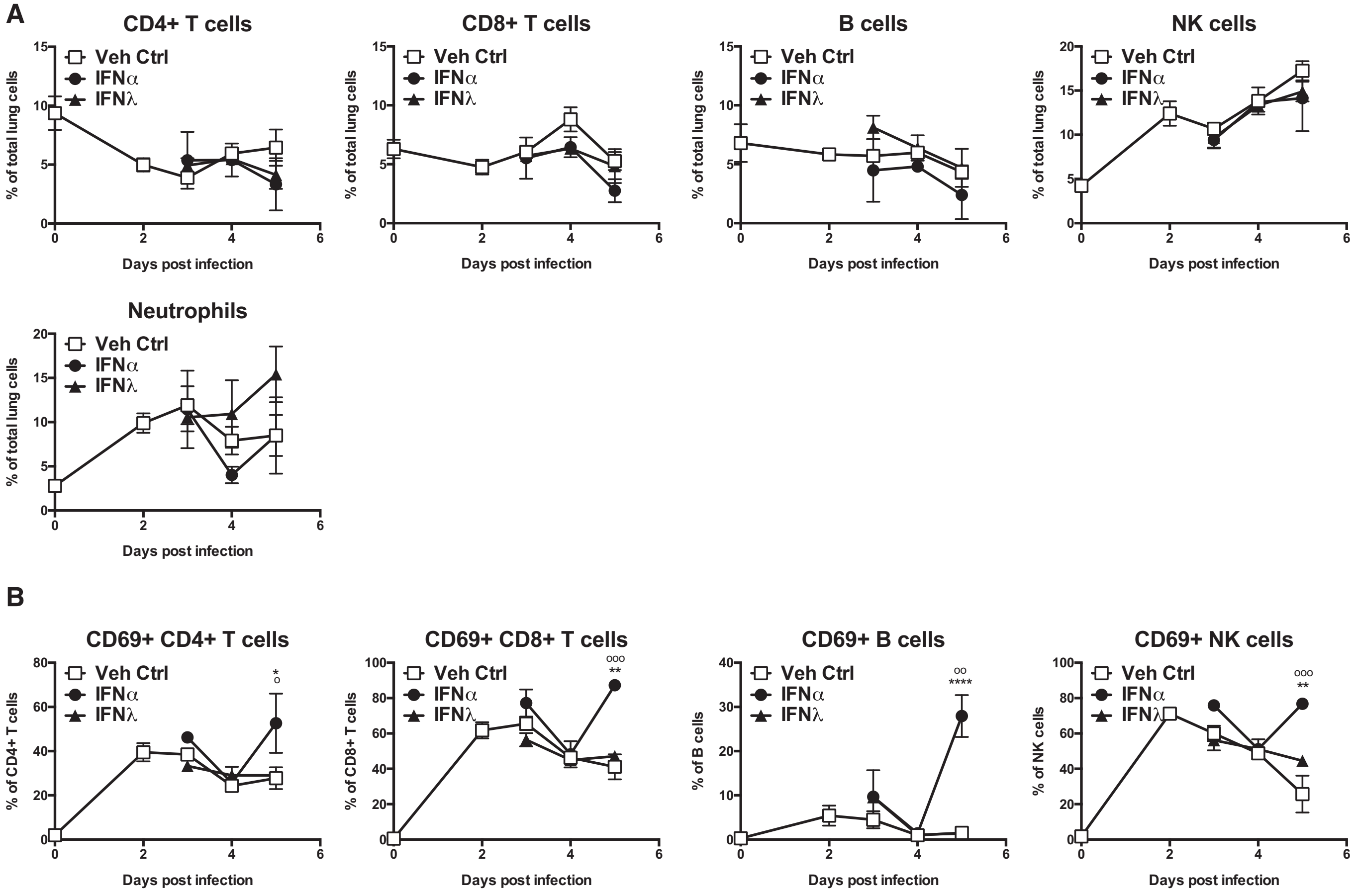

Figure EV2. Immune cell recruitment and lymphocyte activation upon IFNα and IFNλ treatment.

Mice were infected with PR8 and treated with IFNα (circles, 1.45 μg/50 μl), IFNλ (triangles, 2.6 μg/50 μl) or Veh Ctrl (squares, 50 μl PBS). Flow cytometric quantification (A) of lymphocyte subsets and neutrophils and (B) of CD69 expression on lymphocytes in the lung was performed (data are representative of two independent experiments, n = 3–5). Significance assessed by two-way ANOVA where *indicates IFNα:Veh Ctrl and °indicates IFNα:IFNλ. IFNλ:Veh Ctrl was not significant. $CD69^+$ $CD4^+$ T cells: *P = 0.0159, °P = 0.0138. $CD69^+$ $CD8^+$ T cells: **P = 0.002, °°°P = 0.0003. $CD69^+$ B cells: ****P < 0.0001, °°P = 0.0069. $CD69^+$ NK cells: **P = 0.0012, °°°P = 0.0005 (B). Graphs show mean ± SEM.

© 2016 The Authors
