# Supplementary material for: IFNλ is a potent anti‐influenza therapeutic without the inflammatory side effects of IFNα treatment
Source: EMBO Mol Med. 2016 Aug 12;8(9):1099–112. doi: 10.15252/emmm.201606413 (PMC5009813; doi:10.15252/emmm.201606413)
Supplement: Supplementary file 2 — Table EV1 [file EMMM-8-1099-s002.docx]

**Table EV1: IFNα-specific genes**

| **Symbol** | **Fold change([IFNL] vs [mock])** | **Fold change([IFNa4] vs [mock])** |
| --- | --- | --- |
| Gbp2 | 1.2847389 | 3.9855018 |
| Ms4a6d | 1.3229121 | 3.8090258 |
| Cxcl13 | 1.2582576 | 3.7380526 |
| Ifi205 | 1.3771138 | 3.6843872 |
| Gbp2 | 1.2362367 | 3.6796377 |
| Ccl4 | 1.0817877 | 3.5691519 |
| LOC226691 | 1.3775985 | 3.490336 |
| C2 | 1.1232857 | 3.360701 |
| BC049975 | 1.2625252 | 3.3051805 |
| Ccl7 | 1.1421381 | 3.2992876 |
| LOC226690 | 1.1577247 | 3.2811322 |
| 2210415K03Rik | 1.0827683 | 3.1301882 |
| LOC626578 | 1.2755919 | 3.1149218 |
| Ccl8 | 1.2521714 | 3.0029647 |
| Plac8 | 1.1678452 | 2.8865275 |
| Ccl12 | 1.3374633 | 2.7600749 |
| Gvin1 | 1.1066488 | 2.667871 |
| Ifi205 | 1.1526281 | 2.655555 |
| Daxx | 1.3208724 | 2.6530592 |
| Sfrp1 | 1.094101 | 2.6343455 |
| Ccl4 | 1.0771163 | 2.6236436 |
| scl000868.1_2 | 1.1220796 | 2.5899966 |
| Pla1a | 1.17412 | 2.583456 |
| Xdh | 1.286142 | 2.5089364 |
| Nrap | 1.2824109 | 2.496111 |
| Irg1 | 1.2694376 | 2.4912689 |
| LOC381276 | 1.2585015 | 2.4568229 |
| Fcgr1 | 1.1923883 | 2.4304502 |
| Sn | -1.0040914 | 2.4187682 |
| A630077B13Rik | 1.0720068 | 2.3969235 |
| Fcrl3 | 1.0939442 | 2.3719475 |
| Orm1 | 1.2848341 | 2.3607984 |
| Cxcl1 | 1.282936 | 2.3235338 |
| Ccl12 | 1.205247 | 2.309329 |
| Ccr5 | 1.1143029 | 2.258544 |
| Ccr5 | 1.1044458 | 2.2546341 |
| Aif1 | 1.1540902 | 2.2063124 |
| IL1RA | 1.1259896 | 2.1915321 |
| Orm2 | 1.0642031 | 2.1883066 |

**Expanded View Table EV1.** List of genes specifically induced by IFNα treatment of mouse lungs, as assessed by microarray and obtained as explained for Fig 4A and C.
