# Supplementary material for: IFNλ is a potent anti‐influenza therapeutic without the inflammatory side effects of IFNα treatment
Source: EMBO Mol Med. 2016 Aug 12;8(9):1099–112. doi: 10.15252/emmm.201606413 (PMC5009813; doi:10.15252/emmm.201606413)
Supplement: Supplementary file 3 — Table EV2 [file EMMM-8-1099-s003.docx]

**Table EV2: Genes induced by both IFNα and IFNλ**

| **Symbol** | **Fold change([IFNL] vs [mock])** | **Fold change([IFNa4] vs [mock])** |
| --- | --- | --- |
| Apod | 1.2381401 | 30.759808 |
| G1p2 | 12.582239 | 17.87032 |
| Irf7 | 7.9304967 | 16.658989 |
| Cxcl10 | 2.2143095 | 13.8053255 |
| Ifit3 | 7.4381065 | 11.342877 |
| Ifit3 | 7.3490148 | 11.151658 |
| Oas1g | 7.065123 | 10.353304 |
| Ifit3 | 6.983446 | 9.776833 |
| Oas1g | 6.8370776 | 9.515264 |
| Saa3 | 2.5948675 | 9.290621 |
| Ifit2 | 7.3697414 | 8.365278 |
| 2510004L01Rik | 4.9584365 | 7.7792635 |
| Ifi205 | 1.4926294 | 7.6408 |
| Cxcl9 | 1.2995536 | 7.224923 |
| Usp18 | 5.5220284 | 7.197994 |
| Ifit2 | 6.193365 | 7.031815 |
| Usp18 | 5.0637994 | 6.952115 |
| Oasl2 | 5.087273 | 6.873696 |
| Usp18 | 5.2878485 | 6.723355 |
| D14Ertd668e | 2.8958435 | 6.555034 |
| D14Ertd668e | 3.699407 | 6.4160714 |
| Gbp4 | 2.350575 | 6.2309895 |
| Oasl2 | 3.5104594 | 5.784392 |
| Oasl1 | 2.9061675 | 5.7650332 |
| Oas2 | 2.0761542 | 5.656633 |
| Gbp4 | 2.2523344 | 5.63188 |
| 2310061N23Rik | 4.3306913 | 5.550416 |
| LOC240327 | 2.491688 | 5.5120974 |
| LOC380706 | 3.4312286 | 5.385573 |
| Ifi205 | 1.2921005 | 5.384786 |
| Lgals3bp | 2.8075557 | 5.322318 |
| D11Lgp2e | 2.8929808 | 5.290276 |
| Ccl7 | 1.292124 | 5.2692394 |
| D11Lgp2e | 2.8142476 | 5.0043755 |
| Phf11 | 1.6490592 | 4.9638753 |
| Phf11 | 1.6501073 | 4.962559 |
| Igtp | 2.3252106 | 4.9291053 |
| Bst2 | 2.9984183 | 4.9233575 |
| Ms4a6d | 1.571149 | 4.8728056 |
| Mx2 | 2.7230413 | 4.8427873 |
| 2310016F22Rik | 3.7472672 | 4.6708426 |
| Stat1 | 2.6883943 | 4.6326876 |
| Tyki | 2.7527122 | 4.618873 |
| G430091H17Rik | 1.5968853 | 4.4795675 |
| Retnla | 1.7348988 | 4.406877 |
| Plekha4 | 1.37303 | 4.403323 |
| H2-Bf | 1.6390758 | 4.4027514 |
| D630022O22Rik | 1.9958028 | 4.3489933 |
| H2-Q5 | 2.3082147 | 4.3070555 |
| H2-T23 | 2.2825294 | 4.2395864 |
| H2-T23 | 2.1650705 | 4.1895094 |
| H2-T23 | 2.1944022 | 4.104615 |
| LOC215405 | 1.6660186 | 4.0925565 |
| H2-Q8 | 1.585433 | 4.066106 |
| H2-T22 | 1.6271762 | 4.057866 |
| D11Lgp2e | 2.4024088 | 4.040015 |
| Timp1 | 1.8370273 | 4.0019875 |
| Stat2 | 2.278832 | 3.9876232 |
| Lgals9 | 2.6664093 | 3.9612224 |
| H2-T17 | 2.0175605 | 3.9583192 |
| Stat1 | 2.6135163 | 3.9481096 |
| Oasl1 | 2.093974 | 3.9419553 |
| Timp1 | 1.7396249 | 3.911318 |
| Lgals9 | 2.7600443 | 3.8927062 |
| H2-Q8 | 1.5769286 | 3.8447468 |
| EG630499 | 2.0194151 | 3.8295605 |
| H2-T9 | 1.8029337 | 3.8216445 |
| Irgm | 2.1408396 | 3.8143153 |
| AI481100 | 2.2213776 | 3.8029175 |
| D11Ertd759e | 2.443727 | 3.7839625 |
| 0610037M15Rik | 1.7049702 | 3.7430706 |
| LOC223672 | 3.3278382 | 3.635766 |
| Mx1 | 1.8817807 | 3.6175537 |
| 9930016I07Rik | 2.235703 | 3.5322459 |
| Trim30 | 2.5490844 | 3.5084581 |
| H2-Q7 | 1.8880122 | 3.4786675 |
| Ch25h | 1.5598056 | 3.4633694 |
| Hap1 | 2.4412975 | 3.4287496 |
| H2-gs17 | 1.950781 | 3.4092915 |
| H2-Q6 | 1.5298299 | 3.401698 |
| A130019H11Rik | 2.013189 | 3.3149617 |
| Trex1 | 1.6828462 | 3.31025 |
| Mlkl | 1.5810298 | 3.2514563 |
| LOC209387 | 2.3334482 | 3.2385502 |
| H2-K1 | 2.3684187 | 3.2340176 |
| AI481105 | 1.8186089 | 3.209344 |
| H2-Q2 | 1.9600385 | 3.1314785 |
| Lcn2 | 2.0701315 | 3.0385509 |
| Prkr | 2.1289303 | 3.0214646 |
| Parp14 | 1.8764936 | 3.0019128 |
| AI451557 | 1.6489873 | 2.9733593 |
| AA467197 | 1.6216146 | 2.9686012 |
| Ifi47 | 1.7705065 | 2.935394 |
| Slfn1 | 1.4351065 | 2.920352 |
| Tap1 | 1.5016826 | 2.8928645 |
| LOC547343 | 1.7944801 | 2.8723974 |
| Ly6e | 1.9523795 | 2.8541107 |
| Pdcd1lg1 | 1.5818467 | 2.8522847 |
| Ube2l6 | 2.0045724 | 2.8324037 |
| H2-K1 | 1.633641 | 2.8313124 |
| Ifi35 | 1.7652152 | 2.793865 |
| Ly6e | 2.0356948 | 2.7541096 |
| Psmb8 | 1.9470115 | 2.7421615 |
| BC023741 | 1.7080458 | 2.7384381 |
| Ube2l6 | 1.9146559 | 2.7242072 |
| Serpina3n | 1.4873064 | 2.6806304 |
| Trex1 | 1.5035621 | 2.6784012 |
| Zc3hdc1 | 2.0388103 | 2.6482623 |
| Isg20 | 2.0820792 | 2.636252 |
| Ddx58 | 1.7925901 | 2.627278 |
| LOC56628 | 1.6872761 | 2.6137996 |
| Adar | 1.5399421 | 2.6008403 |
| Ifitm1 | 1.7192231 | 2.589983 |
| Isgf3g | 1.7623416 | 2.5368586 |
| Psmb8 | 1.6406516 | 2.5209894 |
| Isgf3g | 1.6602575 | 2.4857764 |
| Ddit3 | 1.4071276 | 2.479593 |
| Mthfd2 | 1.4739643 | 2.4754684 |
| Ube1l | 1.7667987 | 2.4473689 |
| BC006779 | 1.5244318 | 2.4411485 |
| 1600029O10Rik | 1.4329995 | 2.4365482 |
| Slc26a4 | 1.5684803 | 2.429241 |
| Psmb9 | 1.6915319 | 2.4273193 |
| Trafd1 | 1.6403863 | 2.4032388 |
| H2-L | 1.6447854 | 2.3979113 |
| LOC56628 | 1.662748 | 2.3934884 |
| H2-T10 | 1.4309374 | 2.3781328 |
| Gbp6 | 1.2431705 | 2.3724232 |
| H2-D1 | 1.6361959 | 2.3366296 |
| Ly6c | 1.3391235 | 2.2953086 |
| 1500032H18Rik | 1.9219496 | 2.2738466 |
| Trafd1 | 1.3488805 | 2.2672293 |
| AA175286 | 1.8393234 | 2.2619362 |
| Oasl1 | 1.4839362 | 2.238957 |
| LOC435565 | 1.4153317 | 2.2314155 |
| Casp4 | 1.3467966 | 2.2303135 |
| Sp100 | 1.399498 | 2.2287695 |
| Trafd1 | 1.3290308 | 2.2222214 |
| H2-L | 1.615305 | 2.2153995 |
| Ogfr | 1.6525278 | 2.2057378 |
| Psme1 | 1.6406735 | 2.205333 |
| Trim34 | 1.412451 | 2.1985784 |
| Sp100 | 1.5144222 | 2.1783187 |
| Gbp6 | 1.2402816 | 2.1741354 |
| Psmb10 | 1.4112344 | 2.162792 |
| Sp100 | 1.3645018 | 2.1585033 |
| Rtp4 | 1.5687493 | 2.1564984 |
| Ly6a | 1.581486 | 2.1502624 |
| Rgs1 | 1.6049118 | 2.147321 |
| 9830148G24Rik | 1.0573686 | 2.145608 |
| Pml | 1.2997061 | 2.1448886 |
| Ifitm3 | 1.6881515 | 2.143363 |
| Tor3a | 1.6353865 | 2.1273417 |
| LOC327957 | 1.1520467 | 2.1208866 |
| Tapbp | 1.3822311 | 2.117514 |
| Tor3a | 1.6559536 | 2.116158 |
| 2310046K10Rik | 1.4498899 | 2.088682 |
| Pigr | 1.3750216 | 2.0849867 |
| Scotin | 1.6041082 | 2.082178 |
| Psme1 | 1.6118859 | 2.0699055 |
| Cxcl10 | 1.19115 | 2.0564387 |
| Ppa1 | 1.9896586 | 2.0559535 |
| Gbp5 | 1.1213963 | 2.0556252 |
| Grn | 1.1659713 | 2.0365043 |
| Egr1 | 2.3207893 | 2.0331163 |
| Ccr5 | 1.0897138 | 2.0307877 |
| Ccl11 | 1.0220569 | 2.030755 |
| Tspo | 1.4138999 | 2.0288517 |
| Fbxw17 | 1.7944506 | 2.0249305 |
| Oas1b | 1.3740138 | 2.0240252 |
| Tor3a | 1.52482 | 2.0114543 |
| Adar | 1.4522613 | 2.004042 |
| AW011738 | 1.454881 | 2.0030415 |
| Psmb10 | 1.5097951 | 2.0029573 |
| Serpina3g | 1.1966841 | 2.0026345 |
| H2-M3 | 1.2114582 | 2.001266 |
| Slp | 1.1227368 | 1.9987378 |
| Tspo | 1.3297167 | 1.9948919 |
| Serpina3m | 1.2407002 | 1.9946738 |
| Car13 | 1.2493434 | 1.9930084 |
| 2610208M17Rik | 1.1826186 | 1.9905577 |
| 5830484A20Rik | 1.8170404 | 1.9890424 |
| BC006779 | 1.4648407 | 1.9848917 |
| Indo | 1.0053425 | 1.982279 |
| Mpeg1 | 1.674815 | 1.9739152 |
| Trim34 | 1.345098 | 1.9736916 |
| Sirpb1 | 1.286069 | 1.9702507 |
| Sdc3 | 1.0020195 | 1.9660679 |
| Adar | 1.4992374 | 1.9614336 |
| A530060O05Rik | 1.0813787 | 1.9512907 |
| Plekha4 | 1.1195204 | 1.9364331 |
| Irg1 | 1.1428504 | 1.9352313 |
| Ass1 | 1.0815881 | 1.9309489 |
| Tgtp | 1.3699518 | 1.9213822 |
| Ccl2 | 1.1208168 | 1.914876 |
| Lilrb4 | 1.4830303 | 1.9145397 |
| Reg3g | 1.4894595 | 1.904181 |
| Slc15a3 | 1.0781494 | 1.8921789 |
| Slfn2 | 1.1543213 | 1.8915346 |
| Tap2 | 1.4922214 | 1.8899333 |
| 4930599N23Rik | 1.5573094 | 1.8861489 |
| LOC625360 | 1.1335478 | 1.8797626 |
| Sepw1 | 1.0732526 | 1.8795005 |
| Tlr2 | 1.2307844 | 1.8676444 |
| Cd86 | 1.2685654 | 1.8673105 |
| Sct | 1.0748252 | 1.8639894 |
| Pnp | 1.2343369 | 1.8625478 |
| H2-T10 | 1.1165234 | 1.8622842 |
| Slp | 1.2236809 | 1.860513 |
| Aif1 | 1.0947992 | 1.8581631 |
| Lgals9 | 1.6351675 | 1.8571086 |
| Adar | 1.3633056 | 1.855705 |
| Pml | 1.2736572 | 1.8537519 |
| LOC232400 | -1.0202882 | 1.8528591 |
| Selp | 1.193679 | 1.8466377 |
| Ddit3 | 1.3216918 | 1.84197 |
| Clec4a3 | 1.1215587 | 1.8409234 |
| Oas1b | 1.3042043 | 1.8405097 |
| BC022145 | 1.2637795 | 1.8301772 |
| C4 | 1.1586028 | 1.8241267 |
| Nt5c3 | 1.3435187 | 1.8185577 |
| Trim21 | 1.2830515 | 1.8137977 |
| Samhd1 | 1.3491179 | 1.8125848 |
| BC013712 | 1.0485169 | 1.8056426 |
| 1500012F01Rik | 1.177802 | 1.8043287 |
| Gch1 | 1.3020761 | 1.8018657 |
| A430084P05Rik | 1.0487506 | 1.7980877 |
| Ccl5 | 1.1859064 | 1.7969415 |
| Ccl3 | 1.0213332 | 1.7962427 |
| Lag3 | -1.0122541 | 1.795113 |
| Cd72 | 1.0228245 | 1.7893636 |
| Tspo | 1.3455933 | 1.7881826 |
| 2810022L02Rik | 1.7305329 | 1.7873198 |
| 2010012F05Rik | 1.3817141 | 1.7861695 |
| Ankrd1 | 1.35828 | 1.7859273 |
| BC048546 | -1.0308908 | 1.783321 |
| 4930599N23Rik | 1.4105327 | 1.7828981 |
| Scotin | 1.2903026 | 1.7768028 |
| Stat1 | 1.370105 | 1.7751642 |
| 0610009F02Rik | 1.417364 | 1.7741919 |
| Calr | 1.136929 | 1.768203 |
| 2010106G01Rik | 1.2895528 | 1.7681198 |
| Oas3 | 1.2783053 | 1.7643006 |
| Slamf9 | 1.0606245 | 1.7618225 |
| Nkg7 | 1.046285 | 1.7612797 |
| Clic4 | 1.1001037 | 1.7520108 |
| Rgs1 | 1.3436689 | 1.7493767 |
| Tcirg1 | 1.1896461 | 1.7493116 |
| Samhd1 | 1.3238294 | 1.7466183 |
| Cxcl16 | 1.4849741 | 1.7452772 |
| BC094916 | 1.0475341 | 1.7449073 |
| 4933430F08Rik | 1.0456191 | 1.7399868 |
| Tmsb10 | -1.109044 | 1.7395878 |
| 1300018I05Rik | 1.2921277 | 1.7357433 |
| Pira11 | 1.1178164 | 1.7351241 |
| Il4i1 | 1.3522341 | 1.7310942 |
| MGC6357 | 1.25791 | 1.728159 |
| Scotin | 1.2938106 | 1.7276497 |
| B630009B09Rik | 1.1741602 | 1.7174274 |
| LOC383125 | 1.3348019 | 1.7172891 |
| Junb | 1.5211506 | 1.7162735 |
| Rarres2 | 1.1331202 | 1.7158643 |
| C4 | 1.1163961 | 1.7143289 |
| Npc2 | 1.7241905 | 1.7123568 |
| Casp4 | 1.2898856 | 1.7080015 |
| Tnfsf13b | 1.0658611 | 1.6951498 |
| C1qb | 1.2487588 | 1.693105 |
| Noxo1 | 1.0260259 | 1.6867074 |
| Tapbpl | 1.4036711 | 1.6861074 |
| Evi2a | 1.0925378 | 1.6805695 |
| 2010012F05Rik | 1.2136961 | 1.6800739 |
| LOC434484 | 1.1305635 | 1.6793357 |
| Ms4a7 | 1.1007632 | 1.6770626 |
| Tlr13 | 1.2315418 | 1.6765938 |
| Clecsf12 | 1.2596731 | 1.6742636 |
| Ankrd1 | 1.1816775 | 1.6704594 |
| Clecsf12 | 1.2899553 | 1.6698402 |
| Irgm | 1.2928563 | 1.6642164 |
| Ctps | 1.2023996 | 1.6635807 |
| Rnf31 | 1.245605 | 1.6614555 |
| Pla1a | 1.0712485 | 1.6576855 |
| Trim25 | 1.4385402 | 1.657476 |
| Tdrd7 | 1.4526963 | 1.6566073 |
| Relb | 1.1817224 | 1.6548029 |
| Slc7a5 | 1.2470579 | 1.6536175 |
| Ppp1r14d | 1.3804692 | 1.653262 |
| Rrbp1 | 1.312188 | 1.6527531 |
| Smpdl3b | 1.0635484 | 1.6518362 |
| Tcirg1 | 1.1764401 | 1.6516805 |
| Ankfy1 | 1.1793396 | 1.6506516 |
| Tapbp | 1.3068169 | 1.6497266 |
| Ifi204 | 1.1233225 | 1.6461195 |
| scl000868.1_2 | 1.0458633 | 1.6407843 |
| Zfp313 | 1.2273004 | 1.6373821 |
| Zfp313 | 1.3427753 | 1.6275567 |
| Ccl9 | 1.4394734 | 1.6237038 |
| Ccl19 | 1.2698966 | 1.6225989 |
| Fcer1g | 1.104761 | 1.6223042 |
| Il1rn | 1.0506401 | 1.6167262 |
| Tnfsf13b | 1.0558382 | 1.616629 |
| Cd69 | 1.1426711 | 1.6142421 |
| Lst1 | -1.0293639 | 1.6131693 |
| Serping1 | -1.0025697 | 1.6131527 |
| Pols | 1.2167155 | 1.6120976 |
| Cyp7b1 | 1.2756162 | 1.611783 |
| LOC381244 | 1.1534431 | 1.61155 |
| Slc2a6 | 1.2625706 | 1.6107432 |
| C4 | 1.0499246 | 1.6103112 |
| BC023892 | 1.353994 | 1.6065263 |
| Evi2a | 1.0879519 | 1.6011761 |
| Psme2b | 1.3709245 | 1.6005177 |
| Ifitm1 | 1.2950852 | 1.5979434 |
| F830008K13Rik | 1.2544249 | 1.5949384 |
| Serping1 | -1.0440838 | 1.5946349 |
| A430056A10Rik | 1.3266184 | 1.5940015 |
| Ctss | 1.2088864 | 1.5883632 |
| Art3 | 1.0410066 | 1.5868651 |
| LOC240921 | 1.0479943 | 1.5865359 |
| Ms4a8a | 1.1345174 | 1.5861446 |
| Ifitm2 | 1.3166716 | 1.5852414 |
| Cp | 1.2249973 | 1.5790125 |
| 1810009M01Rik | 1.3119202 | 1.5786229 |
| Wisp2 | -1.0884558 | 1.5781375 |
| A130015P11Rik | 1.1788441 | 1.5779599 |
| Ly86 | 1.064914 | 1.5750246 |
| Ctps | 1.3228042 | 1.5746181 |
| Ctps | 1.261551 | 1.5738385 |
| BC024561 | 1.1399192 | 1.5717908 |
| Tinagl | 1.0367861 | 1.570407 |
| Cxcl14 | 1.0903791 | 1.5670463 |
| Xcl1 | -1.0007374 | 1.5644635 |
| Tgfbi | 1.3123162 | 1.5621724 |
| B2m | 1.4298825 | 1.5584313 |
| Mthfd2 | 1.1744696 | 1.5579389 |
| Tgfbi | 1.2293031 | 1.5578811 |
| Atp10a | 1.1806552 | 1.5575788 |
| 1500012F01Rik | 1.1593927 | 1.5565454 |
| A130072J07 | 1.4594407 | 1.5547892 |
| Apobec1 | 1.0573468 | 1.5541328 |
| Hspb6 | 1.0921898 | 1.5537612 |
| Atf3 | 1.3182873 | 1.5506775 |
| Cd180 | 1.0180258 | 1.5485319 |
| BC049354 | 1.1393989 | 1.548339 |
| Irgm | 1.1785004 | 1.5476046 |
| B630009I04Rik | 1.16654 | 1.5465243 |
| BC013672 | 1.2780781 | 1.5458938 |
| Mill2 | 1.2839385 | 1.5445967 |
| Clecsf12 | 1.2993085 | 1.54169 |
| Upp1 | 1.0511423 | 1.5409486 |
| Azi2 | 1.1990424 | 1.5377803 |
| F630107D10Rik | 1.0873708 | 1.5366734 |
| Prkcdbp | 1.1568284 | 1.5361063 |
| Ctsz | 1.1812767 | 1.5360832 |
| Akp2 | 1.0820922 | 1.5346539 |
| LOC381010 | 1.2453744 | 1.5319322 |
| Tmsb10 | -1.0172322 | 1.5313413 |
| Rhoc | 1.0478281 | 1.5307486 |
| Ddx24 | 1.2157705 | 1.5293789 |
| Rarres2 | 1.1477493 | 1.5292376 |
| scl0002116.1_6 | 1.2034056 | 1.5256225 |
| Gsdmdc1 | 1.1996303 | 1.5237099 |
| AI481105 | 1.2891465 | 1.5229447 |
| Tinagl | -1.0254425 | 1.5228773 |
| Axud1 | 1.2855201 | 1.522764 |
| Pla1a | 1.1087766 | 1.5226134 |
| Pbef1 | 1.3799819 | 1.5217743 |
| Plod3 | 1.2406632 | 1.5209503 |
| Ppp1r14d | 1.4666433 | 1.5208132 |
| 1200013B22Rik | 1.3181694 | 1.5204556 |
| Tlr7 | 1.0034118 | 1.5203454 |
| C4 | 1.0964634 | 1.5194741 |
| Itih4 | 1.3737946 | 1.5188125 |
| H2-Q5 | 1.2226307 | 1.5184234 |
| Gadd45g | 1.1974773 | 1.5161738 |
| Thbs1 | 1.3436913 | 1.5134673 |
| Klra7 | 1.0613177 | 1.508586 |
| Ctsb | 1.2983029 | 1.5082626 |
| Enpp4 | 1.2865506 | 1.5081517 |
| Tmem176a | 1.0172911 | 1.506547 |
| Ifi203 | 1.048598 | 1.5026933 |
| Klre1 | 1.0261391 | 1.5026431 |
| Irf5 | -1.0438304 | 1.5022235 |
| Irf1 | 1.0727218 | 1.5019846 |
| 2010005H15Rik | 1.5644674 | 1.4659208 |
| Fos | 2.5179932 | 1.4653891 |
| Tacstd2 | 1.6001923 | 1.3650656 |
| C430002D13Rik | 1.5289665 | 1.3244027 |
| Cyr61 | 1.659218 | 1.1214038 |
| Cyr61 | 1.5165956 | 1.1201091 |
| Dusp1 | 1.8283868 | 1.0965629 |

**Expanded View Table EV2.** List of genes induced similarly by IFNα and IFNλ genes, as assessed by microarray and obtained as explained for Fig 4B and D.
